# Development of resilience indicator traits based on daily step count data for dairy cattle breeding

**DOI:** 10.1186/s12711-022-00713-x

**Published:** 2022-03-14

**Authors:** Marieke Poppe, Han A. Mulder, Mathijs L. van Pelt, Erik Mullaart, Henk Hogeveen, Roel F. Veerkamp

**Affiliations:** 1 Animal Breeding and Genomics, Wageningen University and Research, PO Box 338, 6700 AH Wageningen, The Netherlands; 2 Cooperation CRV, Animal Evaluation Unit, PO Box 454, 6800 AL Arnhem, The Netherlands; 3 CRV BV, Innovation, PO Box 454, 6800 AL Arnhem, The Netherlands; 4 Wageningen University and Research, Business Economics, PO Box 8130, 6700 EW Wageningen, The Netherlands

## Abstract

**Background:**

Resilient animals are minimally affected by disturbances, such as diseases and heat stress, and quickly recover. Daily activity data can potentially indicate resilience, because resilient animals likely keep variations due to disturbances that threat animal homeostasis at a low magnitude. We used daily step count of cows to define resilience indicators based on theory, exploratory analysis and literature, and then investigated if they can be used to genetically improve resilience by estimating heritability and repeatability, and genetic associations with other resilience-related traits, i.e. health traits, longevity, fertility, and body condition score (BCS).

**Results:**

Two groups of resilience indicators were defined: indicators describing (1) mean step count at different lactation stages for individual cows, and (2) fluctuations in step count from individual step count curves. Heritability estimates were highest for resilience indicators describing mean step count, from 0.22 for the 2-week period pre-partum to 0.45 for the whole lactation. High mean step count was consistently, but weakly, genetically correlated with good health, fertility, and longevity, and high BCS. Heritability estimates of resilience indicators describing fluctuations ranged from 0.01 for number of step count drops to 0.15 for the mean of negative residuals from individual curves. Genetic correlations with health traits, longevity, fertility, and BCS were mostly weak, but were moderate and favorable for autocorrelation of residuals (− 0.33 to − 0.44) and number of step count drops (− 0.44 to − 0.56) with hoof health, fertility, and BCS. Resilience indicators describing variability of residuals and mean of negative residuals showed strong genetic correlations with mean step count (0.86 to 0.95, absolute), which suggests that adjustment for step count level is needed. After adjustment, ‘mean of negative residuals’ was highly genetically correlated with hoof health, fertility, and BCS.

**Conclusions:**

Mean step count, autocorrelation and mean of negative residuals showed most potential as resilience indicators based on resilience theory, heritability, and genetic associations with health, fertility, and body condition score. Other resilience indicators were heritable, but had unfavorable genetic correlations with several health traits. This study is an important first step in the exploration of the use of activity data to breed more resilient livestock.

## Background

Cows are exposed to various environmental disturbances that threaten homeostasis throughout their lives, such as pathogens, heat waves, and sudden changes in feed composition. The number of disturbances and their severity are expected to increase in the future. For example, due to climate change the number of extreme weather events will likely increase [1, 2]. Therefore, it is important to improve the resilience of cows, which was defined by Colditz and Hine [3] as their capacity to be minimally affected by disturbances, and if they are affected, to quickly recover. One option to improve the resilience of cows is through genetic selection [4, 5]. The advantage of genetic selection is that it can tackle problems through prevention strategies, rather than through the treatment of stress or disease.

The response of cows to environmental disturbances can often be observed through temporary changes in traits such as milk yield [6, 7] and activity [8, 9]. Therefore, patterns in longitudinal data records, such as daily production, activity, or feed intake data, contain information on their response to many kinds of naturally occurring disturbances. When such data are routinely collected, they provide the potential to derive indicators of resilience for animal breeding.

Several resilience indicators based on longitudinal data have been proposed, which were originally aimed at indicating resilience of ecosystems [10–12]. These resilience indicators were the variance and lag-1 autocorrelation of longitudinal traits. Variance indicates how severely a longitudinal trait fluctuates around its expected value. Resilient animals are not expected to have large fluctuations, and therefore a small variance is an indicator of good resilience. Lag-1 autocorrelation indicates how dependent subsequent records are on each other and therefore how slowly the trait recovers from small natural disturbances. Resilient animals are expected to recover quickly and therefore have low lag-1 autocorrelations [13].

The proposed resilience indicators based on patterns in longitudinal data have been investigated in animals. For example, variance and autocorrelation of daily milk yield data and deviations from expected yield have been shown to be promising indicators to select for better resilience: they are heritable [4, 5] and have favorable genetic correlations with response to actual disturbances [14] and health and longevity traits [4, 5]. Similar indicator traits have been successfully calculated from daily feed intake data in pigs [15, 16] and 4-weekly body weight records in layers [17].

In dairy cattle, the development of resilience indicators for genetic selection has mainly focused on daily milk yield data. However, currently sensors generate daily activity data on a large scale. Activity data are expected to be more directly affected by disturbances than milk yield. Most disturbances will first result in a change in activity, followed by a change in milk yield [8]. In addition, numerous studies have shown that diseases [8, 18–20] and heat stress [9, 21] have an effect on activity traits, such as number of steps per day, lying time, standing time, and eating time. Therefore, longitudinal activity data may provide an excellent opportunity to develop resilience indicators. The aim of this research was to use daily step count data of cows to define potential resilience indicators based on theory, literature and exploratory analysis of relations between step count and diseases, and then to investigate if they can really be used to genetically improve resilience by estimating their heritability and repeatability, and their genetic associations with other resilience-related traits, i.e. health traits, longevity, fertility, and body condition score.

## Methods

This study consists of two parts. In the first part, potential resilience indicators for genetic selection based on theory and data exploration are calculated and their genetic parameters are estimated. In the second part, we assess whether the potential resilience indicators indeed reflect resilience by estimating genetic correlations with traits from current Dutch genetic evaluations that are related to resilience, i.e. udder health, hoof health, ketosis, fertility, longevity, and body condition score.

### Data and data preparation

Most data editing was performed in Python versions 3.6 and 3.8.5 using the NumPy [22], Pandas [23], and Statsmodels [24] packages, and when other languages or packages were used, they are indicated in text.

Step count data were measured by Nedap Smarttag leg accelerometers (Nedap, Groenlo, the Netherlands). Part of the accelerometers measured additional traits, such as lying time and standing time. However, since the number of cows with data was largest for step count, we focused on this trait. The data consisted of 9,472,978 records of daily step count, for 18,622 cows from 86 Dutch farms with automatic milking systems between July 1st 2016 and July 1st 2019. Cows were in parities 0 to 14, but only the cows in the first three parities were selected based on records of calving dates available from CRV (Arnhem, the Netherlands): 9429 cows in parity 1, 8608 cows in parity 2, and 6759 cows in parity 3. Other exclusion criteria were: cows not registered in the herd-book, cows that were less than 87.5% Holstein Friesian, cows that calved before 640, 855, or 1070 days of age for first, second, and third lactation, respectively [25], or cows that had a calving interval between the current and previous lactation shorter than 215 days. In addition, data after 450 days in milk (DIM) were removed, and records measured during estrus (explained later) and those with step counts less than 200 steps per day were also removed because the latter are likely to be due to errors from the device after visual inspection (long periods with consistently the same small number of steps). The remaining number of records was 1,823,789 on 7569 cows in parity 1, 1,735,669 on 6840 cows in parity 2, and 1,295,398 on 5342 cows in parity 3. The data contained 11,086 unique cows.

In addition to the step count data, other data sets were available from CRV to assist in data preparation. The first data set contained milk yield of cows measured during single milk visits to automatic milking systems and conventional milking systems, and these data were used to determine when cows were lactating or dry. From these data, only the data of the cows in the step count data set were selected, i.e. 15,955,347 records on 7568 cows in parity 1, 18,543,964 records on 6840 cows in parity 2, and 17,487,044 records on 5342 cows in parity 3. The second data set contained insemination records and was used as one of two ways to determine when a cow was in estrus (explained in the following paragraph). From the insemination dataset, data on cows from the step count dataset were selected, i.e. 35,149 records on 9971 cows.

Part of the step count records (61%) contained a variable that describes the number of seconds during which the accelerometer was actually measuring during a day, which was usually 86,400 s (whole day). For 0.6% of the records with a known measuring time, the measured time was less than 86,400 s, which means that the device was not working during the whole day. We were interested in complete time series for as many cows as possible, to be able to calculate resilience indicators based on complete step count patterns. Complete time series are especially important for calculating autocorrelations [12]. Therefore, we decided to keep the records with a recording time less than 86,400 s, and to estimate the number of steps for the whole day by multiplying them by 86,400 divided by the time measured. For records with an unknown measuring time, it was assumed that the device did measure during the whole day. Records before or after a period of at least 7 days without records were removed, because it is likely that, on those days, the device was attached to or removed from the leg of the cow and did not measure during the whole day. Finally, based on the step count data, the days that cows were in estrus were determined. Since estrus detection measurements of Nedap (Groenlo, the Netherlands) were not available, a method adapted from Roelofs et al. [26] was used as follows. If the number of steps for a day was larger than the mean for the 10 days before plus 2.5 times the standard deviation for the 10 days before, that day was determined as an estrus day. Estrus could not occur before 14 DIM or when a cow was pregnant. The timing of pregnancy was estimated based on the next calving date minus 278 days (gestation length of Holsteins according to [27]). In addition to the determination of estrus based on step count level, records were classified as estrus records if the cow was inseminated on that day according to the data set with insemination records. Records classified as estrus records were excluded from all analyses, to avoid an effect of estrus on the resilience indicators.

### Calculation of resilience indicators

**Fig. 1:**
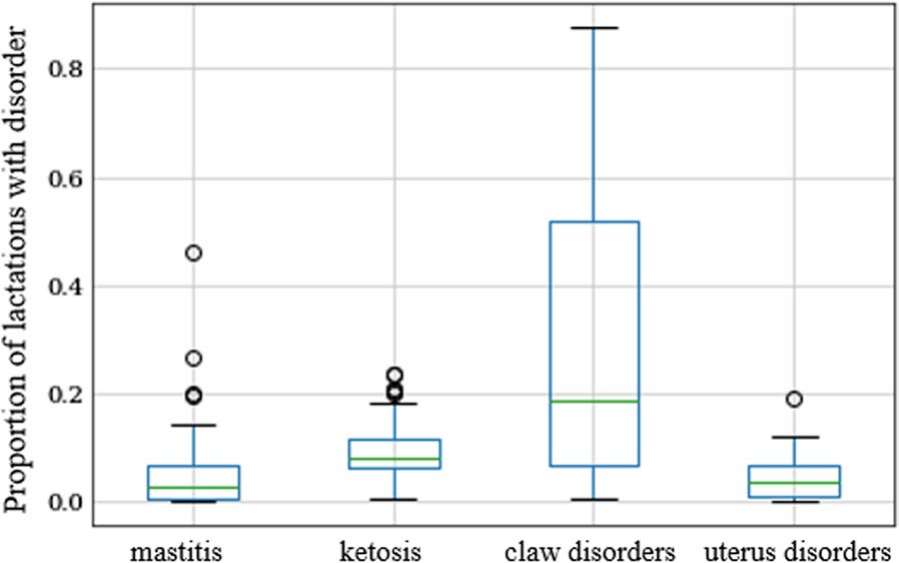
Boxplots of the proportion of lactations with a registration of a disease within farms. Each boxplot includes only the farms with at least one registration of the disease. Only cows in lactations 1, 2, and 3 were included, and the proportion of diseased lactations within each herd was calculated as the number of lactations with a disease registration, divided by the total number of lactations

**Fig. 2:**
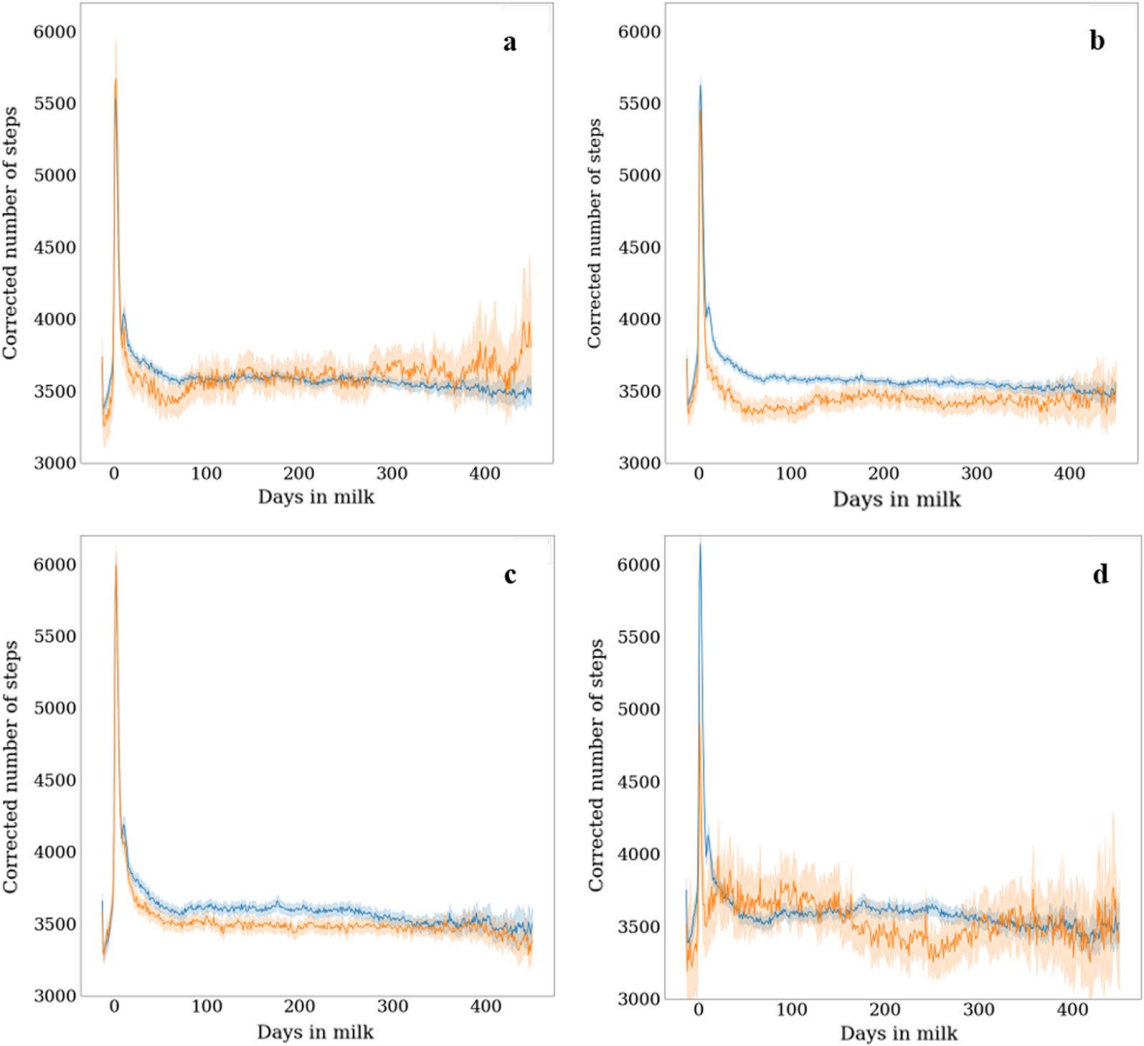
Comparison of step count between lactations with and without registered disease. Step count is corrected for parity, herd-month, and pregnancy status. The mean number of corrected steps is indicated in orange for lactations with a registered disorder, and in blue for lactations without a registered disorder. **a** mastitis, **b** ketosis, **c** claw disorders, and **d** uterus disorders. 95% confidence intervals indicated by lighter areas around the lines

Before calculating resilience indicators, a preliminary phenotypic analysis was performed to investigate how step count differed between cows in lactations 1, 2, and 3 with and without several diseases, i.e. mastitis, ketosis, claw disorders, and uterus disorders. Knowing how step count differs, helps to define traits that could potentially indicate resilience. Disease registrations were available from two sources of data: (1) registrations of clinical mastitis, clinical ketosis, claw disorders, and uterus disorders done by farmers, and professional claw trimmers within the Digiklauw program [28], and (2) milk production registration records, which included indications of mastitis, and indications of clinical or subclinical ketosis based on milk acetone level, milk β-hydroxybutyric acid level, and fat-to-protein ratio. For each lactation with step count records, presence or absence of registration or indication of mastitis, ketosis, claw disorders, and uterus disorders was determined. Since registrations or indications were not available for all the farms, we focused on farms with at least one registration or indication of the disease in question, and with step count data for at least five lactations, which resulted in: 48 farms with mastitis records (30 had farmer registrations and 26 had milk production registrations), and 5.7% of the lactations (1–3) with step count data for these farms had mastitis according to the data; 75 farms with ketosis records (8 had farmer registrations and 75 had milk production recording registrations), and 9.5% of the lactations with step count data for these farms had ketosis; 40 farms with claw disorder registrations, and 31% of the lactations with step count data for these farms had a claw disorder; and 23 farms with uterus disorder registrations, and 4.7% of the lactations with step count data for these farms had a uterus disorder. The proportion of lactations with disorders varied largely between farms, as illustrated in Fig. 1, which may be partly due to registration not being mandatory, which may also cause a lower incidence of diseases than that found in the literature for the Netherlands [29–31], except for ketosis indications from milk production registration [32]. Therefore, in this study these data were used for exploration purposes only, and not for genetic analysis. The data exploration performed on the relation between step count and disease is presented in Fig. 2, which shows number of steps (corrected for parity, herd-month, and pregnancy status) for lactations (1–3) with and without registered diseases on the farms with registrations. Disease seems to coincide with decreased step count, for short or long periods of time and sometimes during particular stages in the lactation. Therefore, two types of resilience indicators were derived from the daily step count data: (1) indicators based on mean step count level at different stages throughout lactation, and (2) indicators based on fluctuations in step count level.

#### Indicators based on mean step count level

**Fig. 3:**
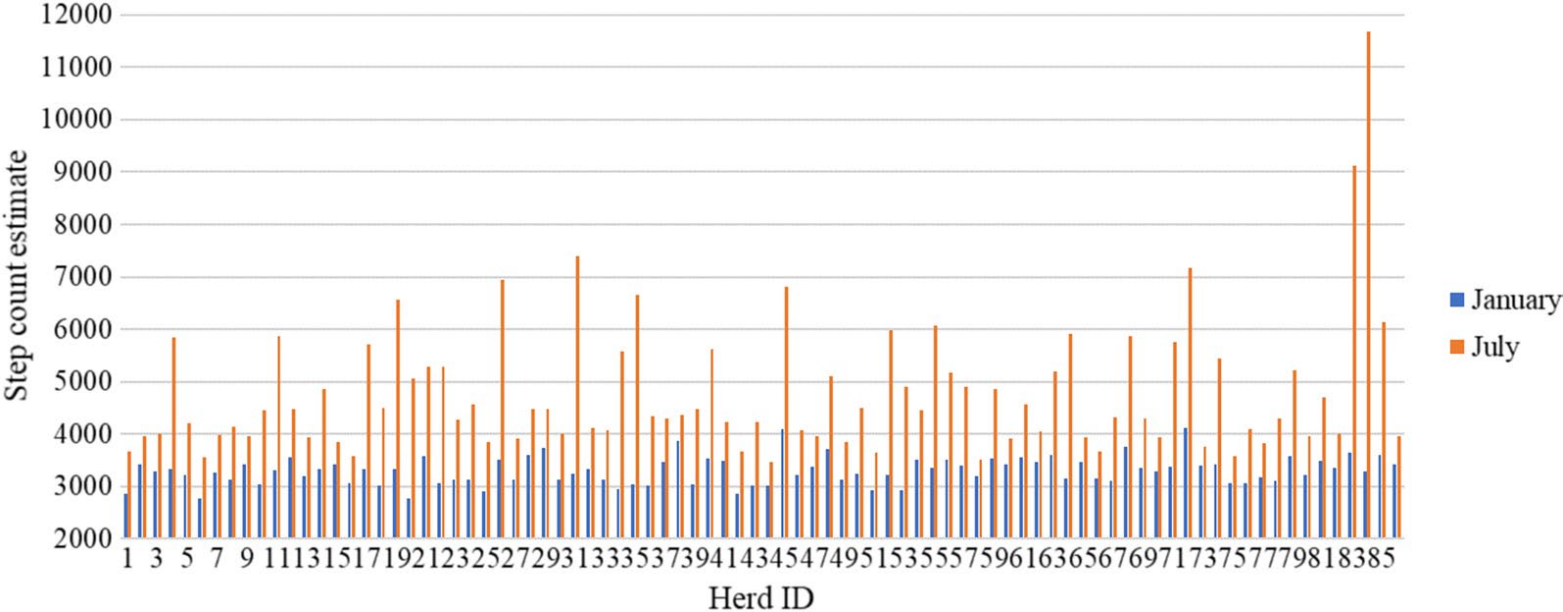
Estimated herd-month effects for all herds for January and July. Estimates are estimated herd-month effects plus intercept (reference: pregnancy status ‘not pregnant’). Effects were estimated for all months of the year, but for clarity only the estimates for January and July are shown

Because of the general association of disturbances with decreased number of steps shown in Fig. 2, mean step count at different stages of the lactation was calculated as a resilience indicator. To avoid differences in step count level between cows due to differences in season and pregnancy status rather than resilience, first a model was fitted to adjust the number of steps per day for these factors as follows:

1 $${y}_{ijk}= H{M}_{j}+pre{g}_{k}+{e}_{ijk},$$

where ${y}_{ijk}$ is a step count record, $H{M}_{j}$ is herd-month $j$ (herd 1 to 86 and month 1 to 12), $pre{g}_{k}$ is pregnancy status $k$ (not pregnant, pregnant, dry, close-up, or unknown), and ${e}_{ijk}$ is the residual. A record was assigned as ‘pregnant’ if it was within 278 days before the next calving date, as “not pregnant” if the next calving date was known but the record was not within 278 days before that date, as “dry” if the record was between the last milking of the lactation and the next calving date, as “close-up” if the record was less than 14 days before the next calving date, and as “unknown” if the next calving date was unknown. The “unknown” class was included, because otherwise cows that were selected for culling after the current lactation or that did not manage to become pregnant would be excluded, which potentially could exclude non-resilient cows. The estimates of herd-month effect from the model are shown in Fig. 3, represented as the herd-month estimate + intercept (pregnancy status ‘not pregnant’). For clarity, only the effects for January and July are shown. Figure 3 shows that, for some herds, the difference between the estimates for January and July was large, while for others it was small, which suggests that some herds may be grazing in summer and some were not. Because the herds were anonymous, no information about grazing, other management practices, or location was known. By correcting for herd-month, we adjusted for any differences in management practices or weather conditions between herds over the year. Differences in management between cows within herds were unknown and thus were not taken into account.

After fitting Model (1) on the data, the residuals ${e}_{ijk}$ were used as the “corrected number of steps”. For each lactation, the mean of the corrected number of steps was calculated for different stages of the lactation: (1) complete lactation: DIM 1–450, (2) early lactation: DIM 1–28, (3) later lactation: DIM 75–450, and (4) pre-partum: DIM − 14 to − 1. The three stages of lactation—early lactation, later lactation, and pre-partum—were selected based on a preliminary analysis that showed different step count levels for different stages of the lactation upon visual inspection (blue lines in Fig. 2), which may mean that step count at different lactation stages contains different information about resilience. A sharp average decline in step count was shown approximately in the first four weeks after calving (also observed by Brzozowska et al. [33]), which may represent adaptation to lactation after calving. Hence this stage was named ‘early lactation’. A steady step count level was observed from ~ 75 DIM onwards, averaged over parities, which may represent the baseline step count level. Hence this stage was named ‘later lactation’. Lower step count levels were observed in the two-week period before calving, and activity in this period may be informative about resilience during the transition period. Hence, this stage was named ‘pre-partum’. The mean number of corrected steps for the whole lactation and for later lactation were only calculated for cows with data for at least four weeks. The mean numbers of corrected steps for early lactation and pre-partum were calculated for cows with data for at least one week (and not 4 weeks as for the other indicators), because these periods consisted of a maximum of 4 and 2 weeks, respectively. After calculating the resilience indicators (mean number of corrected steps at different stages of the lactation), for each parity, outliers of these indicators were removed when values deviated more than 4 times the standard deviation from the mean of all lactations in that parity. The final number of records for each of the resilience indicators based on mean step count per parity are in Table 1.

**Table 1:** Descriptive statistics of potential resilience indicators based on daily step count

Resilience indicator^a^	Number of records parity 1	Number of records parity 2	Number of records parity 3	Mean	SD	Min	Max
Mean complete lactation	6877	6059	4593	10.90	769.63	− 2912.94	3518.49
Mean early lactation	3555	4712	3632	405.50	1142.96	− 3913.73	5803.58
Mean later lactation	6452	5567	4122	− 55.89	741.57	− 3133.34	3143.33
Mean prepartum	2030	4303	3336	− 97.99	1143.46	− 4650.73	4859.06
Mean negative residuals	6800	6005	4524	− 620.29	281.01	− 1876.08	− 157.27
LnVar_steps_	6830	6041	4556	13.18	0.87	10.34	16.28
r_auto_steps_	6825	6042	4555	0.32	0.19	− 0.43	0.89
Number of step count drops	6826	6042	4556	1.39	0.70	0.00	4.11
Mean residuals during step count drops	6816	6023	4534	− 685.34	429.89	− 2528.28	0.00

Step count data were adjusted for herd x month, which explains the low means

^a^LnVar_steps_ = natural log-transformed variance of step residuals; r_auto_steps_ = lag-1 autocorrelation of step residuals; *SD* standard deviation

#### Indicators based on fluctuations in step count

Because disturbances may result in temporary drops in step count (Fig. 2), additional resilience indicators were calculated based on fluctuations in step count level. To be able to study short-term fluctuations that are independent of the long-term trend throughout lactation, first a model was fitted on each individual lactation to adjust for the long-term trend. The same model was used as in Poppe et al. [4] to adjust the daily milk yield for the lactation curve shape. This model is a quantile polynomial regression model with a 0.7 quantile, and it was fitted on the corrected number of steps per day from Model (1) as follows:

2 $${y}_{t}= {\beta }_{0}+ {\beta }_{1}*t+{\beta }_{2}*{t}^{2}+{\beta }_{3}*{t}^{3}+{\beta }_{4}*{t}^{4}+\varepsilon ,$$

where ${y}_{t}$ is the corrected number of steps from Model (1) on DIM $t$, ${t}^{n}$ are DIM to the power of $n$, where $n$ is equal to 1, 2, 3, or 4, ${\beta }_{n}$ are regression coefficients describing the relationships between ${t}^{n}$ and ${y}_{t}$, and $\varepsilon$ is the error term. The quantreg package [34] and the poly function in R 3.5.3 [35] were used. Quantile regression [36] with a quantile of 0.7 was used instead of classical regression to reduce the effect of drops in step count due to disturbances on the expected step count level. This generates a curve that is expected to be close to an unperturbed step count curve. After fitting the step count models, for each cow the residuals from her expected step count curve were calculated as ${y}_{t}-\widehat{{y}_{t}}$. A number of resilience indicators were then calculated from the step count residuals. The first two resilience indicators were equivalent to the resilience indicators developed by Poppe et al. [4] based on milk yield residuals: the natural log-transformed variance (LnVar_steps_) and lag-1 autocorrelation (r_auto_steps_) of the step count residuals. Low LnVar_steps_ and low r_auto_steps_ were expected to indicate good resilience, because of few fluctuations in step count and quick return to the baseline [11, 12]. In addition to LnVar_steps_ and r_auto_steps_, a resilience indicator was calculated that describes the mean of the negative residuals from Model 2 (referred to as the ‘mean of all negative residuals’). Resilient cows are expected to have less extreme negative deviations than less-resilient cows. Furthermore, for each lactation, the number of step count drops with at least 10 negative deviations in a row was calculated, expressed as the number of drops per 100 days (referred to as the ‘number of step count drops’). During such drops, it is likely that the cow had to cope with a disturbance. Step count drops of less than 10 negative deviations (e.g. 5), were also investigated, but they did not seem to have any relationships with diseases based on exploratory phenotypic observations. Therefore, it was decided to base the step count drops on 10 days of negative deviations. Resilient cows are expected to have less step count drops than less-resilient cows. Finally, the mean of the negative residuals during these step count drops was calculated (referred to as the ‘mean of negative residuals during step count drops’). Resilient cows are expected to have less extreme step count drops than less-resilient cows. All of the above-mentioned resilience indicators were calculated only for lactations with data for at least four weeks. After calculating the resilience indicators, for each parity, outliers were removed when values deviated more than 4 times the standard deviation from the mean of all lactations in that parity. The final number of records on the resilience indicators describing fluctuations in step count are in Table 1.

### Analysis

#### Phenotypic exploration of relation between resilience indicators and disease registrations

Phenotypic associations between the resilience indicators and the registered diseases mentioned earlier were explored using an analysis of variance:

3 $${y}_{ijklm}= pa{r}_{i}+{mast}_{j}+{ket}_{k}+{claw}_{l}+{ut}_{m}+{e}_{ijklm},$$

where ${y}_{ijklm}$ is the resilience indicator, $pa{r}_{i}$ is parity $i$ (1 to 3), ${mast}_{j}$ is mastitis group $j$ (0 for no recorded mastitis and 1 for recorded mastitis), ${ket}_{k}$ is ketosis group $k$ (0 for no recorded ketosis and 1 for recorded ketosis), ${claw}_{l}$ is claw disorder group $l$ (0 for no recorded claw disorder and 1 for recorded claw disorder), ${ut}_{m}$ is uterus disorder group $m$ (0 for no recorded uterus disorder and 1 for recorded uterus disorder), and ${e}_{ijklm}$ is the error term. For each disease, only records from herds with registrations of that disease were included, as explained earlier. Effects of mastitis, ketosis, claw disorders, and uterus disorders were considered significant at a p-value of 0.05.

#### Univariate genetic analysis of resilience indicators

A genetic analysis was performed on the resilience indicators using mixed animal models in ASReml 4.1 [37]. The pedigree contained five generations of ancestors. The following univariate repeatability model was used:

4 $$\mathbf{y}=\mathbf{X}\mathbf{b}+{\mathbf{Z}}_{\mathbf{1}}\mathbf{a}+{\mathbf{Z}}_{\mathbf{2}}\mathbf{p}+\mathbf{e},$$

where $\mathbf{y}$ is a vector of repeated records on the resilience indicator in parities 1, 2, and 3; $\mathbf{b}$ is a vector of the fixed effects for the analyzed trait, i.e. parity, age at calving in months nested within parity, year-season of calving nested within parity, herd-year nested within parity, and a covariate describing the first available DIM with a step count record nested within parity; $\mathbf{a}$ is a vector of the additive genetic effects of the cows in $\mathbf{y}$ for the analyzed trait, $\mathbf{a}\sim \mathrm{N}(\mathbf{0},\mathbf{A}{\sigma }_{a}^{2})$ where $\mathbf{A}$ is the additive genetic relationship matrix and ${\sigma }_{a}^{2}$ is the additive genetic variance; $\mathbf{p}$ is a vector of the permanent environmental effects of the cows in $\mathbf{y}$ for the analyzed trait, $\mathbf{p}\sim \mathrm{N}\left(\mathbf{0},\mathbf{I}{\sigma }_{pe}^{2}\right)$ where $\mathbf{I}$ is the identity matrix and ${\sigma }_{pe}^{2}$ is the permanent environmental variance; $\mathbf{e}$ is a vector of the residuals, $\mathbf{e}\sim \mathrm{N}\left(\mathbf{0},\mathbf{I}{\sigma }_{e}^{2}\right)$ where ${\sigma }_{e}^{2}$ is the residual variance. $\mathbf{X}$, ${\mathbf{Z}}_{1}$ and ${\mathbf{Z}}_{2}$ are incidence matrices linking the phenotypic records of the analyzed resilience indicator to the fixed effects and covariates, additive genetic effects, and permanent environmental effects, respectively.

#### Bivariate genetic analysis of resilience indicators

Genetic correlations and permanent environmental correlations among the resilience indicators were estimated using the following bivariate repeatability model:

5 $$\left[\begin{array}{c}{\mathbf{y}}_{\mathbf{1}}\\ {\mathbf{y}}_{\mathbf{2}}\end{array}\right]=\left[\begin{array}{cc}{\mathbf{X}}_{\mathbf{1}}& \mathbf{0}\\ \mathbf{0}& {\mathbf{X}}_{\mathbf{2}}\end{array}\right]\left[\begin{array}{c}{\mathbf{b}}_{\mathbf{1}}\\ {\mathbf{b}}_{\mathbf{2}}\end{array}\right]+\left[\begin{array}{cc}{\mathbf{Z}}_{\mathbf{a}\mathbf{1}}& \mathbf{0}\\ \mathbf{0}& {\mathbf{Z}}_{\mathbf{a}\mathbf{2}}\end{array}\right]\left[\begin{array}{c}{\mathbf{a}}_{\mathbf{1}}\\ {\mathbf{a}}_{\mathbf{2}}\end{array}\right]+ \left[\begin{array}{cc}{\mathbf{Z}}_{\mathbf{p}\mathbf{1}}& \mathbf{0}\\ \mathbf{0}& {\mathbf{Z}}_{\mathbf{p}\mathbf{2}}\end{array}\right]\left[\begin{array}{c}{\mathbf{p}}_{\mathbf{1}}\\ {\mathbf{p}}_{\mathbf{2}}\end{array}\right]+ \left[\begin{array}{c}{\mathbf{e}}_{\mathbf{1}}\\ {\mathbf{e}}_{\mathbf{2}}\end{array}\right],$$

where ${\mathbf{y}}_{\mathbf{i}}$ is a vector of repeated records on a resilience indicator in parities 1, 2, and 3; ${\mathbf{b}}_{\mathbf{i}}$ is a vector of the fixed effects for the trait, which are the same as in the univariate analysis; ${\mathbf{a}}_{\mathbf{i}}$ is a vector of the additive genetic effects of the cows in ${\mathbf{y}}_{\mathbf{i}}$; ${\mathbf{p}}_{\mathbf{i}}$ is a vector of the permanent environmental effects of the cows in ${\mathbf{y}}_{\mathbf{i}}$; ${\mathbf{e}}_{\mathbf{i}}$ is a vector of the residuals. The following assumptions were made about the additive genetic effects, the permanent environmental effects and the residuals:

$$\left[\begin{array}{c}{\mathbf{a}}_{\mathbf{1}}\\ {\mathbf{a}}_{\mathbf{2}}\end{array}\right]\sim \mathrm{N}\left(\left(\begin{array}{c}\mathbf{0}\\ \mathbf{0}\end{array}\right),\mathbf{A} \otimes \left(\begin{array}{cc}{\sigma }_{{a}_{1}}^{2}& {\sigma }_{{a}_{1}{a}_{2}}\\ {\sigma }_{{a}_{1}{a}_{2}}& {\sigma }_{{a}_{2}}^{2}\end{array}\right)\right),$$

$$\left[\begin{array}{c}{\mathbf{p}}_{\mathbf{1}}\\ {\mathbf{p}}_{\mathbf{2}}\end{array}\right]\sim \mathrm{N}\left(\left(\begin{array}{c}\mathbf{0}\\ \mathbf{0}\end{array}\right),\mathbf{I} \otimes \left(\begin{array}{cc}{\sigma }_{{pe}_{1}}^{2}& {\sigma }_{{pe}_{1}p{e}_{2}}\\ {\sigma }_{{pe}_{1}p{e}_{2}}& {\sigma }_{{pe}_{2}}^{2}\end{array}\right)\right),$$

$$\mathrm{and} \quad \left[\begin{array}{c}{\mathbf{e}}_{\mathbf{1}}\\ {\mathbf{e}}_{\mathbf{2}}\end{array}\right]\sim \mathrm{N}\left(\left(\begin{array}{c}\mathbf{0}\\ \mathbf{0}\end{array}\right),\mathbf{I} \otimes \left(\begin{array}{cc}{\sigma }_{{e}_{1}}^{2}& {\sigma }_{{e}_{1}{e}_{2}}\\ {\sigma }_{{e}_{1}{e}_{2}}& {\sigma }_{{e}_{2}}^{2}\end{array}\right)\right),$$

where ${\sigma }_{{a}_{i}}^{2}$ is the additive genetic variance for trait $i$, ${\sigma }_{{a}_{1}{a}_{2}}$ is the additive genetic covariance between two traits, ${\sigma }_{{pe}_{i}}^{2}$ is the permanent environmental variance for trait $i$, ${\sigma }_{{pe}_{1}p{e}_{2}}$ is the permanent environmental covariance between two traits, and ${\sigma }_{{e}_{i}}^{2}$ is the residual variance for trait $i$. ${\mathbf{X}}_{\mathbf{1}}$ and ${\mathbf{X}}_{\mathbf{2}}$, as well as ${\mathbf{Z}}_{\mathbf{a}\mathbf{1}}$ and ${\mathbf{Z}}_{\mathbf{a}\mathbf{2}}$, and ${\mathbf{Z}}_{\mathbf{p}\mathbf{1}}$ and ${\mathbf{Z}}_{\mathbf{p}\mathbf{2}}$ are the incidence matrices linking the phenotypic records of the two analyzed traits to the fixed effects and covariates, additive genetic effects, and permanent environmental effects, respectively. Genetic correlations (${r}_{g}$) and permanent environmental correlations (${r}_{pe}$) were calculated as ${r}_{g}=\frac{{\sigma }_{{a}_{1}{a}_{2}}}{{\sigma }_{{a}_{1}}{\sigma }_{{a}_{2}}}$ and ${r}_{pe}=\frac{{\sigma }_{{pe}_{1}{pe}_{2}}}{{\sigma }_{{pe}_{1}}{\sigma }_{p{e}_{2}}}$.

#### Genetic associations between resilience indicators and health traits, longevity, fertility, and body condition score

Genetic associations between the resilience indicators and several health traits, longevity, fertility, and body condition score were estimated, to obtain more support about whether the resilience indicators really contain information about resilience. Resilient cows are expected to be healthy, live long and be fertile, and have sufficient body condition to cope with disturbances. Genetic correlations with these traits were estimated using the multiple trait across country evaluation (MACE) procedure [38], which requires sire estimated breeding values (EBV) instead of phenotypes to estimate genetic correlations [39–41]. Therefore, it enabled us to explore genetic associations of the resilience indicators with these traits, for which we did not have (a sufficient amount of) phenotypes available for this study, while the official sire EBV are based on phenotypes from the entire Dutch-Flemish population. The sire EBV that we used were the udder health index, hoof health index, ketosis index, fertility index, productive longevity, and body condition score (for clarity, these EBV were based on official genetic evaluations, which included the data used in this study, but the national data was much larger and the overlap has limited impact on the results). The udder health index is based on clinical mastitis registrations of farmers and somatic cell count records in parities 1, 2, and 3 [42]. The hoof health index is based on hoof disorder registrations by professional hoof trimmers in parity 1 and in parity 2 and older, and feet and leg conformation in parity 1 [43]. The ketosis index is based on milk acetone level, milk β-hydroxybutyric acid level, and fat-to-protein ratio on test-days in parities 1 and 2, and parity 3 and older [44]. The fertility index is based on interval between first and last insemination and interval between calving and first insemination, measured in parities 1, 2 and 3 [45]. EBV for productive longevity were based on a random regression on observations for survival in months 1 to 72 after first calving [46]. EBV for body condition score were based on single observations per cow in parity 1, scored by professional type classifiers [47]. High values of the indices and EBV indicate good health, fertility, and longevity, and high body condition score.

As input for the MACE procedure, sire EBV from Cooperation CRV and CRV BV from the official run of December 2020 were used for the health, longevity, fertility and body condition score traits. For the resilience indicators, sire EBV resulting from the univariate analyses were used. EBV for the resilience indicators were required to have a minimum reliability of 10% to be included in the MACE procedure, and sires were required to be born after 1985 and be officially registered as a sire for artificial insemination. Differences in reliability of EBV between sires were accounted for in the MACE procedure by de-regressing the EBV. De-regression adjusts EBV for their reliability and makes the genetic variance in de-regressed proofs constant and independent of the reliability of the EBV, while the total variance of the de-regressed proofs is still a function of the reliability [41, 48]. The number of sires with EBV that were used was larger than 800 (and maximally 1164) for all traits except for number of step count drops (297 sires).

#### Partial genetic correlations

Because LnVar_steps_, mean of all negative residuals, and mean of negative residuals during step count drops were strongly genetically correlated with mean number of steps, for these traits, partial genetic correlations with the health traits, longevity, fertility, and body condition score were calculated. Partial genetic correlations represent the genetic association between these resilience indicators and the health traits, longevity, fertility, and body condition score among cows with the same step count level. Partial genetic correlations (${r}_{xy}{,}_{z}$) between resilience indicators ($x$) and the health traits, longevity, fertility, and body condition score ($y$), adjusted for mean number of steps for the whole lactation ($z$) were calculated as:

6 $${r}_{xy}{,}_{z}=\frac{{r}_{xy}-{r}_{xz}{r}_{yz}}{\sqrt{1-{r}_{xz}^{2}}\sqrt{1-{r}_{yz}^{2}}}.$$

The genetic correlations between the resilience indicators and mean number of steps for the whole lactation were obtained from the bivariate analyses. The other genetic correlations were estimated using the MACE procedure.

## Results

### Descriptive statistics of the resilience indicators

The resilience indicators that describe mean step count consider step count corrected for herd-month and pregnancy stage, and are thus centered to 0. Therefore, means of the corrected number of steps can be negative and such negative values correspond to a mean number of steps smaller than expected based on herd-month and pregnancy stage. Raw step count values were between ~ 3000 and 700 steps per day (Fig. 2). The mean corrected number of steps was on average largest in the first four weeks of lactation (405.5; Table 1) and smallest in the two weeks before calving (− 98.0). In other words, cows had on average 503.5 (405.5 + 98.0) steps per day more in the first four weeks after calving than in the two weeks before calving. The mean corrected number of steps was on average − 55.89 steps per day from DIM 75 onwards and 10.90 per day across the whole lactation. The mean of all negative residuals from the lactation-specific models was on average -620.3 steps. LnVar_steps_ was on average 13.2 and r_auto_steps_ was on average 0.32. On average, 1.39 step count drops occurred per 100 days, and the mean of the negative residuals during these drops was on average − 685.34.

### Phenotypic associations between diseases and resilience indicators

None of the resilience indicators had a significant association with mastitis (Table 2). However, lactations with a ketosis registration had a significantly lower mean step count throughout lactation after calving, less extreme negative residuals throughout lactation, lower LnVar_steps_, and less extreme negative residuals during step count drops than lactations without a ketosis registration. Lactations with a claw disorder registration had a significantly lower mean step count for later lactation and the whole lactation, less extreme negative residuals throughout lactation, lower LnVar_steps_, lower r_auto_steps_, and less extreme negative residuals during step count drops than lactations without a claw disorder registration. Lactations with a uterus disorder registration had a significantly lower mean step count for early lactation, less extreme negative residuals throughout lactation, lower LnVar_steps_ and r_auto_steps_, and less step count drops than lactations without a uterus disorder registration. Mean steps pre-partum was the only trait without a significant association with any of the diseases.

**Table 2:** Effect of diseases on the resilience indicators based on daily step count

Resilience indicator^a^	Effect of mastitis (SE)	P-value	Effect of ketosis (SE)	P-value	Effect of claw disorder (SE)	P-value	Effect of uterus disorder (SE)	P-value
Mean complete lactation	6.85 (35.49)	0.85	− 137.86 (38.51)	0.00	− 182.36 (27.57)	0.00	− 31.85 (52.91)	0.55
Mean early lactation	− 4.91 (57.89)	0.93	− 267.20 (64.04)	0.00	− 37.93 (49.64)	0.45	− 235.69 (103.69)	0.02
Mean later lactation	20.15 (36.63)	0.58	− 142.78 (40.41)	0.00	− 188.96 (28.06)	0.00	13.50 (53.67)	0.80
Mean prepartum	66.40 (76.78)	0.39	− 41.60 (81.71)	0.61	− 4.38 (65.13)	0.95	− 181.36 (144.50)	0.21
Mean negative residuals	15.19 (12.87)	0.24	51.40 (14.05)	0.00	80.10 (10.05)	0.00	44.16 (19.31)	0.02
LnVar_steps_	− 0.024 (0.038)	0.53	− 0.20 (0.042)	0.00	− 0.25 (0.030)	0.00	− 0.17 (0.057)	0.00
r_auto_steps_	0.0086 (0.009)	0.32	− 0.0047 (0.009)	0.62	− 0.015 (0.007)	0.03	− 0.054 (0.013)	0.00
Number of step count drops	0.025 (0.032)	0.45	− 0.035 (0.035)	0.32	0.0080 (0.025)	0.75	− 0.11 (0.049)	0.02
Mean residuals during step count drops	8.84 (20.14)	0.66	97.09 (21.99)	0.00	87.94 (15.72)	0.00	60.27 (30.13)	0.05

^a^LnVar_steps_ = natural log-transformed variance of step residuals; r_auto_steps_ = lag-1 autocorrelation of step residuals

### Genetic analysis

#### Heritabilities and repeatabilities

Table 3 shows estimates of variance components, heritabilities, and repeatabilities of all resilience indicators. The heritability and repeatability estimates were highest for the traits describing means of corrected number of steps at different stages of the lactation, and ranged from 0.22 and 0.39, respectively, for the mean corrected steps prepartum, to 0.45 and 0.74, respectively, for the mean corrected steps during the whole lactation. For the other resilience indicators, the heritabilities ranged from 0.01 for the number of step count drops to 0.15 for the mean of negative deviations, and the repeatabilities ranged from 0.03 to 0.37.

**Table 3:** Estimates (SE) of genetic parameters from the univariate analyses of the resilience indicators based on daily step count

Resilience indicator^a^	${\sigma }_{a}^{2}$	${\sigma }_{pe}^{2}$	${\sigma }_{e}^{2}$	$r$	${h}^{2}$
Mean complete lactation	223,780 (16,406)	372,050 (8596)	130,859 (2287)	0.74 (0.0060)	0.45 (0.027)
Mean early lactation	261,591 (31,631)	468,060 (18,124)	503,072 (11,624)	0.48 (0.013)	0.27 (0.030)
Mean later lactation	204,592 (16,097)	349,870 (8366)	128,566 (2396)	0.73 (0.0060)	0.43 (0.029)
Mean prepartum	221,480 (34,552)	385,560 (20,767)	602,422 (16,203)	0.39 (0.017)	0.22 (0.033)
Mean negative residuals	4578.21 (682.30)	11,302 (439.26)	19,678.1 (338.64)	0.37 (0.011)	0.15 (0.021)
LnVar_steps_	0.050 (0.0078)	0.13 (0.0051)	0.24 (0.0041)	0.35 (0.012)	0.14 (0.020)
r_auto_steps_	0.00091 (0.00025)	0.0026 (0.00030)	0.022 (0.00036)	0.11 (0.012)	0.037 (0.010)
Number of step count drops	0.005 (0.002)	0.012 (0.0050)	0.37 (0.0060)	0.033 (0.012)	0.012 (0.006)
Mean residuals during step count drops	5205.14 (1317.76)	18,390 (1367.90)	84,593.5 (1449.51)	0.18 (0.013)	0.051 (0.013)

${\sigma }_{a}^{2}$ = additive genetic variance, ${\sigma }_{pe}^{2}$ = permanent environmental variance, ${\sigma }_{e}^{2}$ = error variance, $r$=repeatability, ${h}^{2}$=heritability

^a^LnVar_steps_ = natural log-transformed variance of step residuals, r_auto_steps_ = lag-1 autocorrelation of step residuals

#### Genetic and permanent environmental correlations among resilience indicators

The four traits related to mean corrected number of steps at different stages of the lactation were all strongly genetically correlated with each other (Table 4). The weakest genetic correlation was 0.80 between mean corrected steps prepartum and mean corrected steps in early lactation, and the strongest one was 1.00 between mean corrected steps during the whole lactation and mean corrected steps in later lactation, which is a part-whole relationship. The traits that describe means of corrected steps were also strongly genetically correlated with most traits that describe step count fluctuations, namely LnVar_steps_ (${r}_{g}$ from 0.65 to 0.94), mean of all negative residuals (${r}_{g}$ from − 0.79 to − 0.93), and mean of negative residuals during step count drops (${r}_{g}$ from − 0.85 to − 0.95). These strong genetic correlations suggest that cows with a high mean step count, genetically, tend to have more extreme negative deviations throughout lactation and during step count drops, and higher variability in step count than cows with a low mean step count. Among the traits that describe step count fluctuations, the genetic correlations were strongest between mean of all negative residuals and LnVar_steps_ (− 0.93), between mean of negative residuals during step count drops and LnVar_steps_ (− 0.93), between r_auto_steps_ and number of step count drops (0.94), and between mean of negative residuals during step count drops and mean of all negative residuals (0.96). The remaining genetic correlations were weaker, ranging from − 0.73 between number of step count drops and mean of negative residuals during step count drops, to 0.48 between LnVar_steps_ and number of step count drops. Most permanent environmental correlations had the same sign as the corresponding genetic correlations, but were weaker. Only the permanent environmental correlations of the number of step count drops with other traits were very different from the corresponding genetic correlations with sometimes a different sign, but with large standard errors. In summary, many resilience indicators were strongly genetically correlated between each other and genetic selection on only one of them will therefore change many others.

**Table 4:** Genetic (above diagonal) and permanent environmental (below diagonal) correlations (SE) among the resilience indicators based on daily step count

	Mean complete lactation	Mean early lactation	Mean later lactation	Mean prepartum	Mean negative residuals	LnVar_steps_	r_auto_steps_	Number of step count drops	Mean residuals during step count drops
Mean complete lactation		0.96 (0.013)	1.00 (0.0005)	0.91 (0.037)	− 0.93 (0.025)	0.86 (0.034)	0.25 (0.12)	0.26 (0.18)	− 0.95 (0.049)
Mean early lactation	0.87 (0.023)		0.94 (0.019)	0.80 (0.055)	− 0.88 (0.039)	0.94 (0.032)	0.25 (0.13)	0.21 (0.20)	− 0.92 (0.063)
Mean later lactation	0.99 (0.0012)	0.80 (0.033)		0.92 (0.035)	− 0.93 (0.026)	0.86 (0.036)	0.23 (0.12)	0.33 (0.18)	− 0.93 (0.049)
Mean prepartum	0.73 (0.052)	0.57 (0.074)	0.70 (0.054)		− 0.79 (0.065)	0.65 (0.084)	0.13 (0.15)	0.24 (0.22)	− 0.85 (0.079)
Mean negative residuals	− 0.75 (0.028)	− 0.65 (0.046)	− 0.76 (0.027)	− 0.63 (0.073)		− 0.93 (0.022)	− 0.40 (0.13)	− 0.65 (0.17)	0.96 (0.037)
LnVar_steps_	0.67 (0.035)	0.71 (0.041)	0.64 (0.036)	0.67 (0.076)	− 0.83 (0.023)		0.36 (0.13)	0.48 (0.18)	− 0.93 (0.041)
r_auto_steps_	0.0079 (0.090)	0.045 (0.11)	0.091 (0.089)	0.26 (0.14)	− 0.14 (0.094)	0.00090 (0.10)		0.94 (0.14)	− 0.50 (0.14)
Number of step count drops	− 0.28 (0.17)	− 0.20 (0.20)	− 0.21 (0.17)	− 0.21 (0.25)	0.24 (0.20)	− 0.44 (0.25)	0.78 (0.20)		− 0.73 (0.16)
Mean residuals during step count drops	− 0.65 (0.061)	− 0.69 (0.071)	− 0.75 (0.052)	− 0.66 (0.097)	1.00 (0.061)	− 0.70 (0.057)	− 0.46 (0.090)	− 0.29 (0.17)	

LnVar_steps_ = natural log-transformed variance of step residuals, r_auto_steps_ = lag-1 autocorrelation of step residuals

#### Genetic associations with diseases, longevity, fertility, and body condition score

Most resilience indicators had weak or negligible genetic correlations with the health traits, longevity, fertility, and body condition score (Table 5). However, r_auto_steps_ and the number of step count drops had moderate genetic correlations with hoof health, fertility, and body condition score, ranging from − 0.33 to − 0.44 for r_auto_steps_ and from − 0.44 to − 0.56 for number of step count drops. These genetic correlations mean that cows with a genetically low autocorrelation or a small number of step count drops often had genetically good hoof health and fertility, and a high body condition score. Although the genetic correlations between the resilience indicators based on mean step count and the health traits, longevity, fertility, and body condition score were weak, they were all favorable (from 0.021 to 0.22). This means that a high step count level, especially during lactation and not before calving, was genetically related with good functionality, and particularly good hoof health, little ketosis, good longevity, and a high body condition score. LnVar_steps_ also had consistently positive genetic correlations with the health traits, longevity, fertility, and body condition score, which means that high LnVar_steps_ was genetically associated with good health and functionality. The partial genetic correlations (Table 6) suggest that among the cows with the same step count level, LnVar_steps_ was still positively genetically associated with the other traits, but at a weaker level. Mean of all negative residuals and mean of negative residuals during step count drops had mainly negative and weak genetic correlations with the health traits, longevity, fertility, and body condition score (Table 5). However, most partial genetic correlations (Table 6) were considerably larger in magnitude than the original correlations and the largest ones were positive. In particular, between Tables 5 and 6, the difference in the genetic correlation between mean residuals during step count drops and body condition score was especially large. This was due to the strong negative genetic correlation (− 0.95; Table 4) between mean residuals during step count drops and mean step count, which means that mean of residuals during step count drops was largely determined by step count level. Mean step count itself had a positive genetic correlation with body condition score, which cancelled out the genetic correlation between mean residuals during step count drops and body condition score not explained by step count level. The results from Table 6 suggest that among the cows with the same step count level, those with smaller negative deviations from the expected step count had genetically better health and fertility and a higher body condition score than those with larger negative deviations. In summary, genetic associations of resilience indicators with health and functionality were observed, and the strongest genetic correlations were shown for r_auto_steps_ and number of step count drops with hoof health, fertility, and body condition score.

**Table 5:** Genetic correlations between resilience indicators based on daily step count^a^ and other traits^b^, estimated using multiple trait across country evaluation

	UH	HH	KET	LON	FER	BCS
Mean complete lactation	0.015	0.16	0.20	0.15	0.062	0.17
Mean early lactation	0.025	0.17	0.18	0.22	0.067	0.20
Mean later lactation	0.033	0.16	0.17	0.13	0.065	0.17
Mean prepartum	0.11	0.021	0.036	0.061	0.061	0.029
Mean negative residuals	− 0.047	0.021	− 0.23	− 0.13	0.051	− 0.034
LnVar_steps_	0.11	0.16	0.24	0.12	0.056	0.12
r_auto_steps_	− 0.16	− 0.33	0.0019	0.0022	− 0.44	− 0.34
Number of step count drops	− 0.063	− 0.56	− 0.24	0.16	− 0.44	− 0.56
Mean residuals during step count drops	− 0.031	− 0.044	− 0.11	− 0.14	0.053	0.057

^a^LnVar_steps_ = natural log-transformed variance of step residuals, r_auto_steps_ = lag-1 autocorrelation of step residuals

^b^UH = udder health, HH = hoof health, KET = ketosis resistance, LON = longevity, FER = fertility, BCS = body condition score

**Table 6:** Partial genetic correlations between resilience indicators based on daily step count^a^ and other traits^b^, adjusted for mean number of steps

	UH	HH	KET	LON	FER	BCS
Mean negative residuals	− 0.090	0.47	− 0.12	0.026	0.30	0.34
LnVar_steps_	0.19	0.04	0.14	− 0.018	0.0053	− 0.052
Mean residuals during step count drops	− 0.054	0.35	0.26	0.0081	0.36	0.71

^a^LnVar_steps_ = natural log-transformed variance of step residuals

^b^UH = udder health, HH = hoof health, KET = ketosis resistance, LON = longevity, FER = fertility, BCS = body condition score

## Discussion

The aims of this study were to (1) define potential resilience indicators based on daily step count data according to theory, literature, and data exploration, and (2) to investigate their usefulness for genetically improving resilience by estimating heritability and genetic associations with other resilience-related traits, i.e. health traits, longevity, fertility, and body condition score. Traits that describe the mean number of steps per day at different stages of the lactation were selected based on the assumption that resilient cows do not show many severe declines in step count (Fig. 2; [8, 9]) and therefore have a high step count level. Traits that describe fluctuations in number of steps between days were selected based on the assumption that resilient cows keep, at a low magnitude, variations due to disturbances which threat animal homeostasis and therefore do not show many drops and large fluctuations in step count and recover quickly [10, 11, 13]. The resilience indicators that describe mean step count had a moderately high heritability, and those that describe fluctuations in step count had a lower heritability. Genetic correlations of the step count level traits with health traits, longevity, fertility, and body condition score were all favorable but weak. This means that mean step count can easily be increased through genetic selection, but will coincide with limited improvement of health, longevity, fertility, and body condition score. However, genetic correlations of number of step count drops and r_auto_steps_ with health traits, longevity, fertility, and body condition score were moderately strong and favorable.

This study is one of the first to investigate the heritability of step count level and other step count traits calculated from sensor data in cattle not focused on estrus or fertility. One other study that investigated the heritability of activity traits outside estrus, reported a much lower heritability of mean activity than we did [49]. These authors found a heritability of 0.05 and 0.03 for mean activity index based on head and neck movements, recorded in Israeli Holsteins by electronic activity neck tags in the two-week non-estrus period before and after successful insemination, respectively. The difference between the estimated heritabilities in their study and ours may be explained by differences between the activity trait we measured (step count measured by leg accelerometers from Nedap (Groenlo, the Netherlands) and the activity index based on head and neck movements measured by neck accelerometers from SCR (Netanya, Israel), and between the populations studied. The difference in the length of the measurement period (up to 450 days versus 2 weeks) could also play a role, but this is likely not the main cause: our trait ‘mean step count pre-partum’ was also based on a two-week period, but its heritability was much higher (0.22). Furthermore, Schöpke and Weigel [49] found a heritability of 0.03 for the standard deviation of daily activity, which is much lower than the heritability of the similar trait LnVar_steps_ in this study. However, for this trait Schöpke and Weigel [49] included measurements during estrus, while in our study they were excluded. In addition, they took the standard deviation of the raw data, while we first adjusted for general trend across lactation. Furthermore, differences in trait definition and population studied likely play a role. Because of the moderate to moderately high heritability for the traits calculated from step count data in our study, these traits offer great opportunity for genetic selection, potentially for the benefit of improving resilience and possibly also for other benefits.

In this study, we performed a genetic analysis, while most studies on the development of resilience indicators from activity data are phenotypic analyses (e.g. [20, 50]). However, genetic analysis has a useful advantage over phenotypic analysis, which is that it is not necessary that the animal itself has enough data on response to disturbances to be able to predict its resilience to the following disturbances, as is the case for phenotypic prediction. It has been shown that predicting resilience to a major disturbance using activity data before that disturbance is challenging [50]. With genetic analysis, it is possible to use patterns in activity data on relatives or on animals with shared single nucleotide polymorphisms (SNPs). Such animals with a similar genetic background offer data on response in activity to a wide variety of disturbances, and together give a general picture of the genetic merit for resilience. Therefore, based on genetic analysis, it is possible to estimate the genetic merit for resilience of an animal without data on response to disturbances, when they have family members or animals with shared genotypes that do have data on response to many types of disturbances.

Step count traits are heritable and can be changed through genetic selection, but the question is whether these traits can serve as a proxy to select more resilient cows. According to resilience theory, low variance and autocorrelation of longitudinal traits that are sensitive to disturbances indicate good resilience [10]. Activity is indeed sensitive to disturbances [8, 9, 51], which supports the hypothesis that variance and autocorrelation of step count data contain information on resilience. Moreover, low variability of other traits that are sensitive to disturbances, such as milk yield [4, 5, 14], and daily feed intake in pigs [15, 16] and 4-weekly body weight records in layers [17] has already been shown to be genetically associated with good resilience. Therefore, selection for lower LnVar_steps_ and r_auto_steps_ is expected to result in more resilient cows. For r_auto_steps_, this assumption is supported by its strong positive genetic correlation with number of step count drops and because low values were genetically correlated with good hoof health and fertility and high body condition score. However, for LnVar_steps_ it is important to note that low values were genetically correlated with poor instead of good health, even when adjusted for step count level, although these correlations were weak. It is possible that LnVar_steps_ is associated with other aspects of resilience that are not covered by the existing traits, such as strength of response to disturbances, or response to disturbances other than diseases. However, it is necessary to investigate if this is true, and it is important to consider that selection for lower LnVar_steps_ will coincide with an undesired increase in incidence of health problems. For the trait ‘number of step count drops’, it is intuitively clear that selection for lower values will result in better resilience, and this is also supported by its moderately strong genetic correlation with hoof health, fertility and body condition score. However, because of its very low heritability and its strong genetic correlation with r_auto_steps_, it is more efficient to select for r_auto_steps_ rather than number of step count drops. For the traits that describe mean step count, the hypothesis was that a low step count level indicates poor resilience, because most disturbances, including the diseases explored in Fig. 2 and Table 2, will decrease activity [8, 9]. Indeed, low step count was genetically related with poor health, fertility, and body condition score, albeit weakly. However, it is important to consider that the step count level of a cow is probably not only associated with resilience, but also with the personality of the cow. Therefore, traits that describe fluctuations in step count are probably more directly related with resilience than traits that describe mean step count. Nevertheless, it is worthwhile to investigate the association between mean step count and resilience further, because it offers great opportunity for genetic selection because of its high heritability. For the traits that describe mean of negative residuals throughout lactation and during step count drops, it was hypothesized that more extreme residuals (low values) represent poor resilience, which is rather intuitive. However, the unfavorable genetic correlations with the health traits, but favorable partial genetic correlations suggest that a statistical scale effect [52] is present, where higher mean automatically coincides with higher deviations from the mean. Therefore, these traits should be adjusted for step count level to be useful as resilience indicators. In summary, selection for r_auto_steps_ and number of step count drops steps is likely to result in better resilience. Mean of negative residuals and mean step count are promising resilience indicators as well, but need an adjustment for step count level or more research into the biology of the trait.

The positive genetic correlations of LnVar_steps_ with the health traits, longevity, fertility, and body condition score were surprising. They are likely not, or not entirely, due to a statistical scale effect, because although LnVar_steps_ was strongly genetically correlated with mean step count, the partial genetic correlations adjusted for mean step count were still generally positive. Another explanation is that a very stable step count is not necessarily associated with good resilience, because of the existence of a lower step count limit. Cows have a minimum distance to walk each day to get milked and fed. When severely challenged, for example by a claw disorder, they will likely function at this minimum activity level for a certain time period. Step count will then be very stable, but this is a sign of reduced resilience rather than good resilience. Other studies found varying phenotypic associations of variability of activity with health and resilience traits, ranging from positive [20, 53] to negative [19] associations. The association between LnVar_steps_ and resilience may be curvilinear, where an optimum exists for intermediate LnVar_steps_ values. A curvilinear relationship was also found between the log-transformed variance of clutch size in great tits and fitness, where an intermediate variance was related to the highest fitness [54]. The existence of an optimum level of LnVar_steps_ should be investigated further before it can be used as a resilience indicator.

This study provides important insights into the heritability of resilience indicators from daily step count data and associations with other traits related to resilience that are already in the breeding goal. However, the most important question to be answered before the new traits can be used in practice is how the new resilience indicators are associated with aspects of resilience not covered by the already existing traits. Resilience is a broad concept which is difficult to capture by a single measure [13, 55], and selection for current health traits and longevity already helps to improve resilience. However, health traits consider resilience only to a limited number of diseases and they only include incidence of disease and not severity or recovery rate. Longevity could be considered as a resultant of resilience to all types of disturbances [56], but it is also a resultant of traits not related to resilience, such as productivity. The resilience indicators proposed in this study could offer additional information about resilience, in particular strength of response to disturbances and recovery rate and response to additional disturbances such as heat stress. However, this additional information needs to be investigated first, for example through a validation study similar to Poppe et al. [14] for resilience indicators based on daily milk yield. Furthermore, even if the proposed resilience indicators do not include additional information about resilience compared to the health traits investigated in this paper, r_auto_steps_ and the number of step count drops may offer opportunities to improve hoof health in countries where claw disorders are not recorded. Claw health recording is still performed in a limited number of countries [57, 58], while step count data is increasingly becoming available [59, 60]. Having evidence on how step count data can be used to breed for resilience or more specific health traits is relevant.

Furthermore, the added value of resilience indicators based on daily step count data compared to daily milk yield data, which is more widely available, should be investigated. We hypothesize that resilience indicators based on step count data should be regarded as complementary to resilience indicators based on milk yield, and not as a replacement. While both attempt to serve as indicators of ‘general resilience’ to many types of disturbances, it is impossible for a single longitudinal trait to be sensitive to all types of disturbances [55, 61]. Therefore, a combination of traits is needed to reflect general resilience as well as possible [3]. Indeed, sensitivity to particular disturbances differs for milk yield and step count. For example, step count seems more sensitive to locomotion problems than milk yield, while milk yield seems more sensitive to udder health problems, as suggested by the genetic correlations with claw health and udder health in this study and Poppe et al. [4]. Together, the resilience indicators based on step count data and based on milk yield data will generate a more complete picture of resilience to a wider variety of disturbances than any of them alone. Furthermore, exploitation of simultaneous response profiles in milk yield and step count could offer additional tools to indicate resilience. Similarly, Ben Abdelkrim et al. [55] have explored milk yield and body weight data and showed that 24% of the detected milk yield perturbations coincided with a body weight perturbation, giving additional support that these detected perturbations were really due to a disturbance. In addition, simultaneously tracking perturbations of multiple measures may help to identify differences in coping mechanisms between cows [55]. The benefit of combining multiple longitudinal traits has also been shown when multiple profiles were used to obtain a robust quantification of degree of infection of mastitis [62].

In summary, more research is needed on the added value of the new resilience indicators compared to existing traits related to resilience. However, this study provides an important first step for genetic selection for resilience using activity data.

## Conclusions

This study investigated potential resilience indicators based on daily step count data for genetic selection, based on theory, literature and data exploration. The most promising resilience indicators were autocorrelation, mean step count, and mean of negative residuals from individual curves. Autocorrelation had considerable and favorable genetic correlations with other resilience-related traits (health, fertility, body condition score) but a low heritability (0.04). Mean step count had only weak but favorable genetic correlations with all other resilience-related traits, and a moderately high heritability (0.22 to 0.45). The mean of negative residuals from individual curves had a heritability of 0.15, but needs an adjustment for step count level to have considerable and favorable genetic correlations with other resilience-related traits and thus to be informative about resilience. This research is an important first step in the exploration of the use of activity data for breeding for resilience.

## Data Availability

The data that support the findings of this study are available from Nedap (Groenlo, the Netherlands) and CRV (Arnhem, the Netherlands), but restrictions apply to the availability of these data, which were used under license for the current study, and thus are not publicly available. Data are however available from the authors upon reasonable request and with permission of Nedap (Groenlo, the Netherlands) and CRV (Arnhem, the Netherlands).
